# Persistence to antihypertensive drug classes: A cohort study using the Swedish Primary Care Cardiovascular Database (SPCCD)

**DOI:** 10.1097/MD.0000000000004908

**Published:** 2016-10-07

**Authors:** Miriam Qvarnström, Thomas Kahan, Helle Kieler, Lena Brandt, Jan Hasselström, Kristina Bengtsson Boström, Karin Manhem, Per Hjerpe, Björn Wettermark

**Affiliations:** a Department of Medicine, Center for Pharmacoepidemiology, Karolinska Institutet; b Department of Clinical Sciences, Danderyd Hospital, Division of Cardiovascular Medicine, Karolinska Institutet; c Department of Neurobiology, Care Sciences and Society, Center for Family and Community Medicine, Karolinska Institutet, Stockholm; d Närhälsan R&D Primary Care, R&D-Center Skaraborg, Skövde; e Department of Molecular and Clinical Medicine/Cardiology, Institute of Medicine, Sahlgrenska University Hospital, Sahlgrenska Academy, University of Gothenburg, Gothenburg; f Department of Healthcare Development, Public Healthcare Services Committee, Stockholm County Council, Stockholm, Sweden.

**Keywords:** discontinuation, drug therapy, hypertension, medication persistence, primary healthcare, sex, socioeconomic factors

## Abstract

The aim was to study persistence to, and switching between, antihypertensive drug classes and to determine factors associated with poor persistence.

This was an observational cohort study. The Swedish Primary Care Cardiovascular Database includes data from medical records, socioeconomic data, filled prescriptions, and hospitalizations from national registries for 75,000 patients with hypertension. Patients included in the study were initiated on antihypertensive drug treatment in primary healthcare in 2006 to 2007. We defined class persistence as the proportion remaining on the initial drug class, including 30 days of gap. Patients with a filled prescription of another antihypertensive drug class after discontinuation of the initial drug, including 30 days of gap, were classified as switchers. Persistence to the various drug classes were compared with that for diuretics.

We identified 4997 patients (mean age 60 ± 12 years in men and 63 ± 13 years in women). Out of these, 95 (2%) filled their first prescription for fixed combination therapy and 4902 (98%) for monotherapy, including angiotensin converting enzyme inhibitors (37%), angiotensin receptor blockers (4%), beta blockers (21%), calcium channel blockers (8%), and diuretics (28%). Persistence to the initial drug class was 57% after 1 year and 43% after 2 years. There were no differences in persistence between diuretics and any of the other antihypertensive drug classes, after adjustment for confounders. Discontinuation (all adjusted) was more common in men (*P* = 0.004), younger patients (*P* < 0.001), those with mild systolic blood pressure elevation (*P* < 0.001), and patients born outside the Nordic countries (*P* < 0.001). Among 1295 patients who switched drug class after their first prescription, only 21% had a blood pressure recorded before the switch occurred; and out them 69% still had high blood pressures.

In conclusion, there appears to be no difference in drug class persistence between diuretics and other major antihypertensive drug classes, when factors known to be associated with poor persistence are taken into account.

## 1 Introduction

There is extensive evidence that antihypertensive treatment reduces the risk of cardiovascular morbidity and mortality.^[1]^ Although blood pressure control has improved over the years, many patients with hypertension still do not reach treatment target.^[2–4]^ There are many reasons behind poor blood pressure control despite being treated; for example, inadequate dosing, few different drug classes combined, inadequate monitoring after initiation of treatment, and poor medication taking behavior. Without the appropriate duration and continuity of antihypertensive drug therapy, patients will not benefit from treatment.

There are a number of ways of assessing if patients are using the prescribed medicine and all methods have their own strengths and limitations. Methods used are questionnaires, interviews, pharmacy claims (pharmacy dispensing data), directly observed therapy, pill count, electronic monitoring, and drug or biomarker measurement in body fluids. All these methods can be used to measure adherence/compliance, that is, to the act of conforming to the recommendations made by the provider with respect to timing, dosage, and frequency of medication taking.^[5]^ However, for medication persistence, that is, the duration of time from initiation to discontinuation of therapy^[5]^ most of the methods mentioned above have their limitations. Consequently, longitudinal analyses of pharmacy claims data have been suggested as the “golden standard” in analyses of persistence.^[6]^ For antihypertensive drug treatment, this may be assessed either as class persistence, that is, the proportion remaining on treatment with the drug class used for initiation, or as therapy persistence, that is, the proportion remaining on any antihypertensive treatment. Many previous studies comparing persistence between different antihypertensive drug classes have shown lower persistence with diuretics or beta blockers.^[7–13]^ However, these studies have limitations in their design. Some have used prescription data for antihypertensive drugs without a confirmed diagnosis of hypertension in the individual patient,^[12,13]^ while others have included data only on issued prescriptions and not the actual filled prescriptions by the patients.^[10]^ Furthermore, few previous studies included data on important patient characteristics such as comorbidity, blood pressure before initiating drug treatment, educational level, country of birth, or income.^[7–10]^ These patient factors may all be associated with differences in persistence, and are thus important to include in the analyses to minimize confounding. In a previous study on therapy persistence, where we observed persistence to any antihypertensive treatment, we found that men, younger patients, those with mild-to-moderate systolic blood pressure elevation, and patients born abroad had lower therapy persistence.^[14]^ However, in this study, we want to observe if there are differences in persistence to diuretics compared to other antihypertensive drug classes and to see if the same patient characteristics are also of importance when studying class persistence. Therefore, we determined class persistence to the major antihypertensive drug classes, and assessed associations between persistence and patient age, sex, comorbidity, number of other drugs, baseline blood pressure, and socioeconomic factors. Secondly, we aimed to study patterns of switching between antihypertensive drug classes after initiation.

## 2 Methods

### 2.1 Study population and design

In this observational cohort study based on data from the Swedish Primary Care Cardiovascular Database (SPCCD), we compared persistence to various antihypertensive drug treatments (angiotensin converting enzyme inhibitors, angiotensin receptor blockers [ARBs], beta blockers, calcium channel blockers [CCBs], and fixed combination therapy), to that of diuretics. The SPCCD contains electronic medical records for approximately 75,000 patients with a recorded diagnosis of hypertension in 48 Swedish primary healthcare centers between 2001 and 2008.^[15]^ The medical records data are linked to national registers on filled prescription drugs, hospitalizations, outpatient hospital consultations, deaths, migration, and socioeconomy, using the unique identifiers of each patient.

The study population comprised all patients age 30 years or older with a recorded diagnosis of hypertension (ICD-10 code I10) before the first prescription of an antihypertensive drug. These patients initiated treatment between January 1, 2006 and December 31, 2008. Patient characteristics included age, sex, systolic and diastolic blood pressures, comorbidity, educational level, income, country of birth, and antihypertensive drug class. Cardiovascular comorbidity was defined as a diagnosis of (with corresponding ICD-10 codes) atrial fibrillation (I48, including arterial flutter), heart failure (I50), ischemic heart disease (I20–25), previous stroke/transitory ischemic attack (I60–69, G45), or diabetes mellitus (E10–11), as documented in the medical records and/or the National Patient Register from 2001 and until the date of the first prescription. Blood pressure values assessed were those last recorded prior to the first prescription of an antihypertensive drug. The level of education was classified into 3 groups: primary (9 years or less), lower secondary (<2 years), and upper secondary (3 years or more). Income was classified into 2 groups over or under the median annual income for this cohort in Swedish crowns (SEK, SEK 100 = EUR 10.7, February 2016). Country of birth was classified as Sweden, other Nordic countries, Europe outside the Nordic countries, and other. Antihypertensive drug classes assessed (with corresponding Anatomic Therapeutic Chemical classification system [ATC] codes) were ACE-inhibitors (C09A), ARBs (C09C), beta blockers (C07), CCBs (C08), diuretics (C03A, C03B, C03D, C03E), and fixed combination therapy (CO7FB02, C09BA, C09D). Mineralocorticoid receptor antagonists and amiloride were considered as diuretics.

### 2.2 Class persistence and discontinuation

We analyzed all filled prescriptions of antihypertensive drugs prescribed to the cohort within a 2-year period. Class persistence was defined as the proportion remaining on a specific antihypertensive drug therapy after 1 and 2 years of follow-up, respectively. In this definition, a 30-day gap was included; that is, patients were allowed to be without medication up to a maximum of 30 days and still considered as persistent to the initial antihypertensive treatment. Initiation date was defined as the day (between January 1, 2006 and December 31, 2008) when the first antihypertensive drug prescription was filled. Patients filling a prescription of another antihypertensive drug after running out of tablets from the first initiated drug class or fixed combination therapy and before the end of a 30-day gap were classified as switchers. Patients who filled a new prescription more than 30 days after the end of supply from the former prescription were considered to have discontinued treatment, and therefore not persistent. Due to the potential risk of misclassification of patients combining various drugs, patients who filled a prescription of a drug belonging to another drug class or combination therapy before the end of supply of the initial drug were not considered as switchers.

The definition of class persistence was considered in relation to the Swedish reimbursement regulations with each prescription being valid for 1 year, generally with a time window of 3 months between 2 filled prescriptions. There is a uniform reimbursement system for all Swedish citizens covering drugs prescribed in ambulatory care.^[16]^ The patient pays the full price for subsidized pharmaceuticals included up to a certain level, and then reductions are obtained for the additional cost. The maximum total amount paid by each patient during a 12-month period (out-of-pocket ceiling) for subsidized pharmaceuticals was SEK 1800 (EUR 191) at the time of the study. No fixed co-payments or prescription fees are used within the Swedish reimbursement system. There were no changes in the co-payment levels during the study period, but the reimbursement agency restricted reimbursement for some antihypertensive drugs during the follow-up period.^[17]^

Information on prescribed dosage was extracted from the dosage text through the Swedish Prescribed Drug Register.^[18]^ We used manual reading of the dosage text and developed algorithms in SAS version 9.2 (SAS Institute, Cary, NC) for automatic extraction from the text in order to calculate how long the tablets would last. These algorithms were used for automatic reading of the dosage texts after iterative editing of the algorithms. Of the 106,505 prescriptions, there were 623 (0.6%) prescriptions with no information on dosage instructions and for these prescriptions we assumed 1 tablet per day. The start of the consumption of drugs was assumed to be on the same day as the prescription was filled. When several prescriptions of the same ATC code and strength were filled on the same day they were assumed to be consumed successively. If a new prescription was filled before the end of supply of the previous filled prescription, the remaining tablets were accumulated to the latter one.

### 2.3 Statistical analyses

Data are presented as mean values ± standard deviation or 95% confidence intervals (CIs), and proportions, as appropriate. Persistence was calculated by Kaplan–Meier survival analysis, determining the time of discontinuation for each patient. Patients were censored at death (n = 24), if hospitalized for more than 21 days (n = 138), if initiated on drugs in unit bags (n = 27), or at the end of follow-up (n = 2028). Crude estimates of time to discontinuation were assessed for the potential predictors of class persistence including age, sex, systolic and diastolic blood pressure, cardiovascular comorbidity, number of other drugs, educational level, country of birth, and income. In addition, Cox proportional hazard regressions were used to estimate crude and adjusted hazard ratios together with 95% CIs for discontinuation by all potential predictors mentioned above as well as antihypertensive drug class and fixed combination therapy. A 2-tailed probability value (*P*) of <0.05 was considered significant. All analyses were conducted using SAS version 9.2 (SAS Institute, Cary, NC).

The Regional Ethical Review Board in Gothenburg approved of the study, and written consent from all primary healthcare centers was obtained.

## 3 Results

### 3.1 Patient characteristics

We identified 5225 patients newly initiated on antihypertensive drug treatment during 2006 to 2007. Of these, 64 patients were excluded because they did not fill their prescription at the pharmacy and 164 because they were initiated on combination therapy with more than 1 tablet of antihypertensive treatment. Thus, 4997 patients (mean age 60 ± 12 years in men and 63 ± 13 years in women) were included in the analyses. Patient characteristics are presented in Table 1. Prescriptions of fixed combination therapy were filled for the first time by 95 patients (2%) and monotherapy by 4902 patients (98%), including ACE-inhibitors (37%), ARB (4%), beta blockers (21%), CCB (8%), and diuretics (28%). A first prescription of ACE-inhibitors was to a larger extent filled by men than women (*P* < 0.001) and by patients with diabetes (*P* < 0.001). Women filled their first prescription on diuretics and beta blockers more often than men (*P* = 0.012 and *P* < 0.001, respectively). The ARBs were more often filled by patients with high income and education (*P* = 0.006 and *P* = 0.015, respectively). Further details are found in Table 2.

**Table 1:**
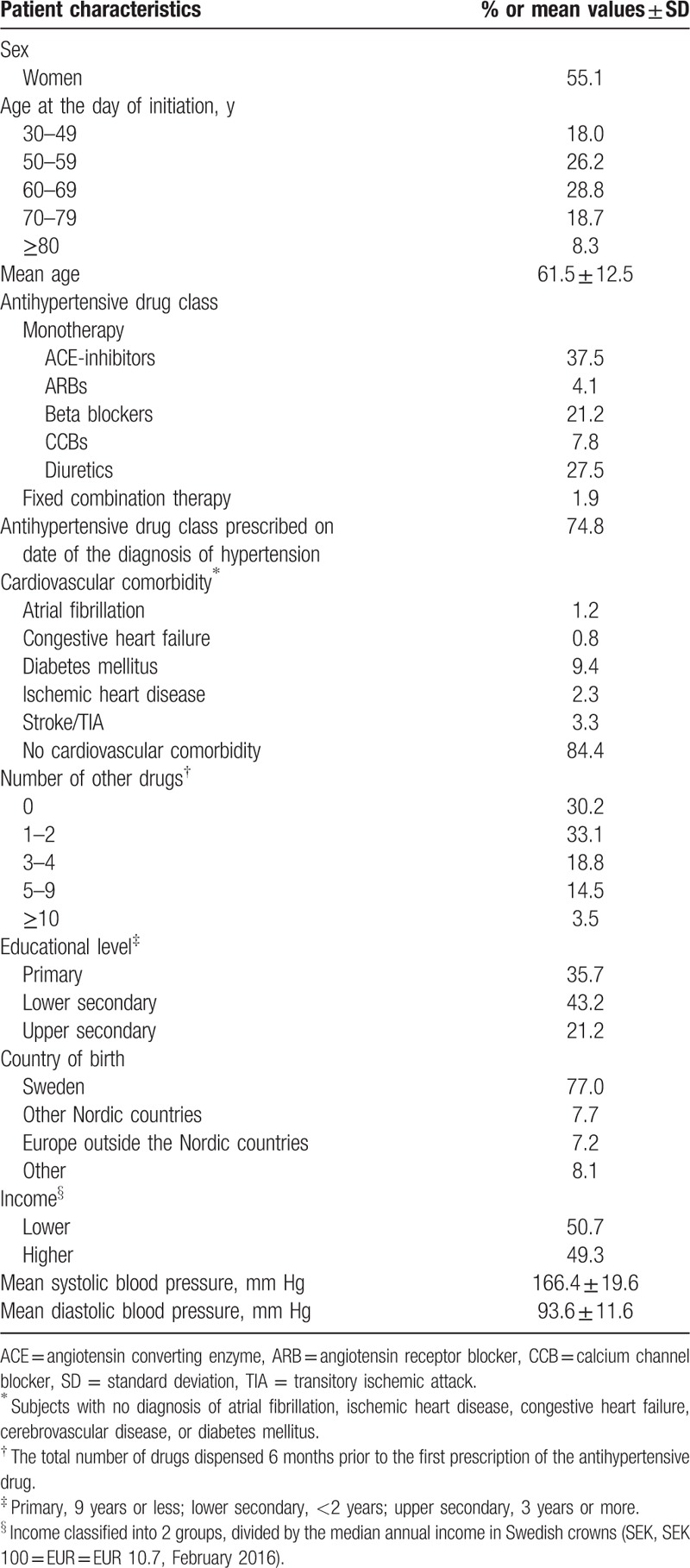
Baseline patient characteristics among 4997 patients with hypertension.

**Table 2:**
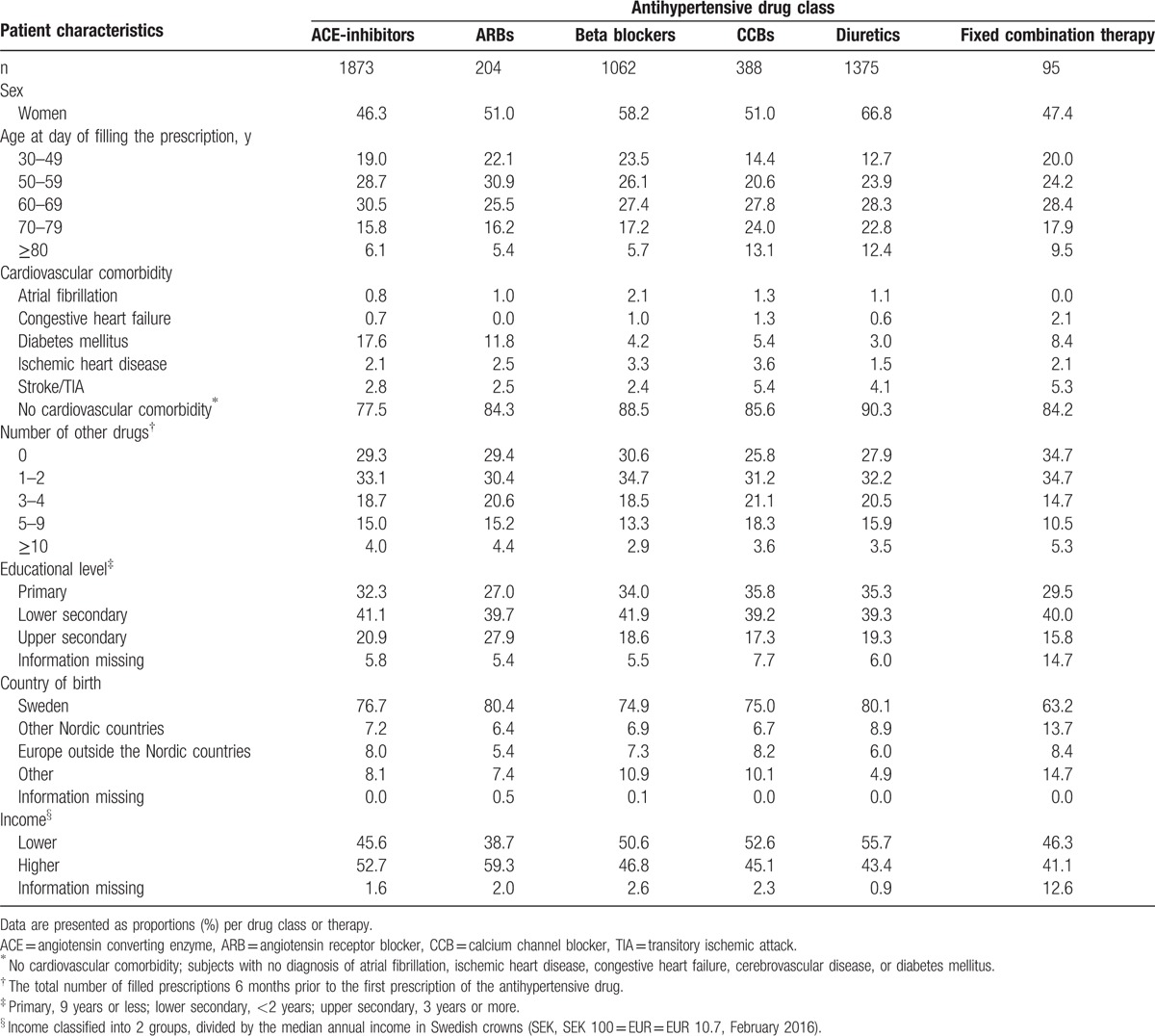
Baseline patient characteristics among 4997 patients with hypertension in primary healthcare according to the antihypertensive drug class or fixed combination therapy.

A total of 4806 patients (96%) had their blood pressure recorded before their first filled prescription. Mean systolic blood pressure was 166 ± 19 mm Hg in men and 167 ± 20 mm Hg in women. Mean diastolic blood pressure was 95 ± 12 mm Hg in men and 92 ± 11 mm Hg in women. Compared to monotherapy, patients initiated on fixed combinations had higher systolic blood pressure (166 ± 20 vs 171 ± 23 mm Hg, *P* = 0.03), but there were no major difference in systolic blood pressures between patients initiated on the different antihypertensive drug classes before they received their first prescription (Table 3).

**Table 3:**
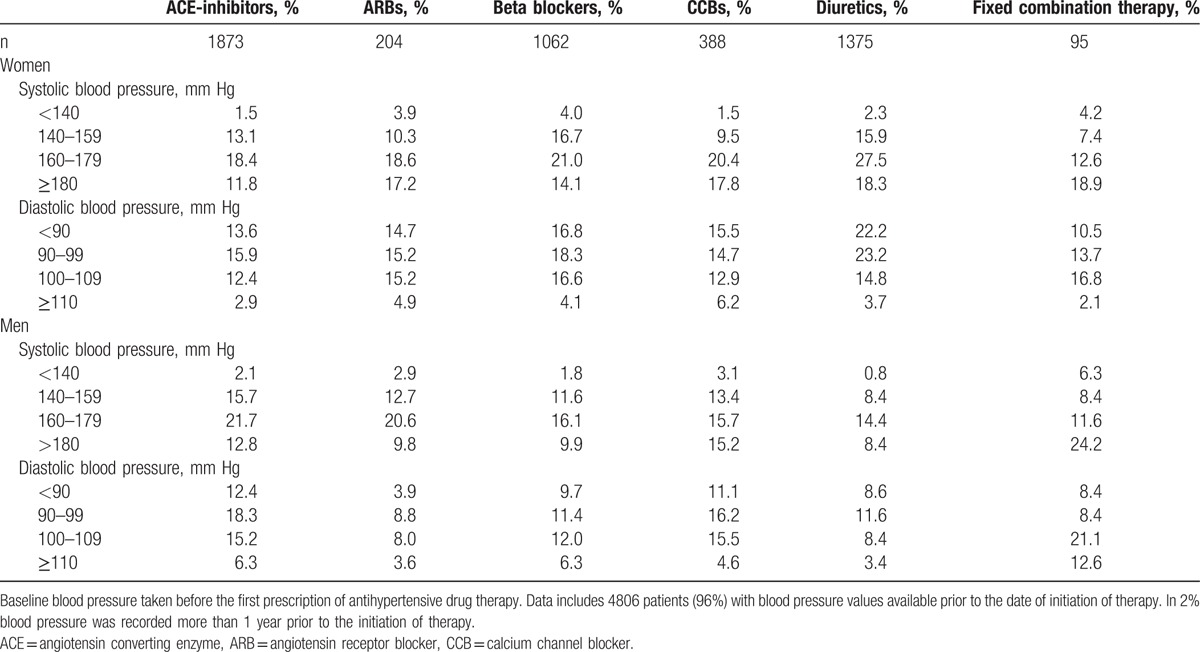
Baseline blood pressure levels according to initiated antihypertensive drug class in 4997 patients.

### 3.2 Class persistence and discontinuation

**Figure 1:**
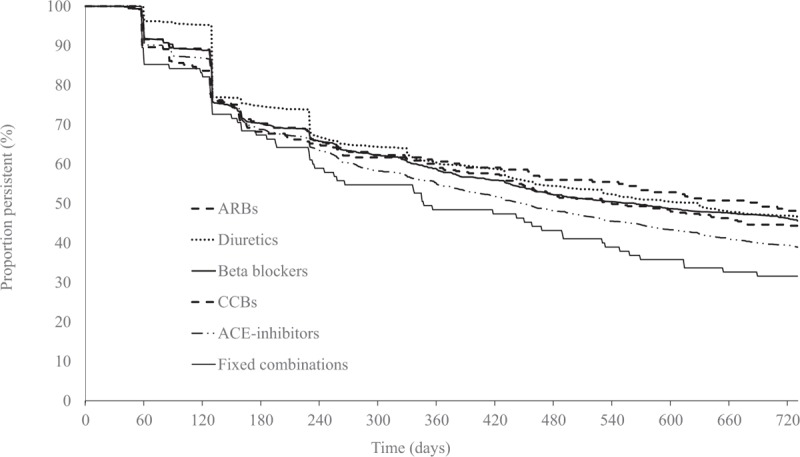
Persistence (unadjusted) according to antihypertensive drug class during 2 years of follow-up in 4997 patients with hypertension in primary healthcare initiated on therapy 2006 to 2007. ACE = angiotensin converting enzyme, ARB = angiotensin receptor blocker, CCB = calcium channel blocker.

**Figure 2:**
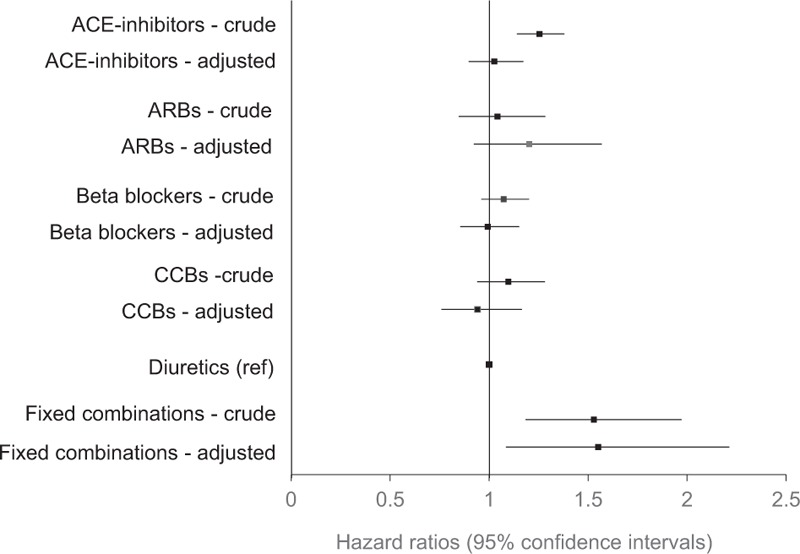
Discontinuation of antihypertensive drug classes 2 years after initiation in 4997 patients with hypertension in primary healthcare initiated on therapy 2006 to 2007. Unadjusted and adjusted hazard ratios. Covariates adjusted for were age, sex, systolic and diastolic blood pressure, diabetes mellitus, no cardiovascular comorbidity (no atrial fibrillation, congestive heart failure, diabetes mellitus, cerebral vascular disease or ischemic heart disease, total number of drugs, income, country of birth, educational level, and initiated drug class). ACE = angiotensin converting enzyme, ARB = angiotensin receptor blocker, CCB = calcium channel blocker.

The overall class persistence during the first year was 57% (men 56%, women 58%). A further 14% discontinued treatment with their initial drug class during the second year. Consequently, the proportion remaining on the initial treatment was 43% (men 42%, women 44%) after 2 years. Kaplan–Meier plots for the individual drug classes are shown in Fig. 1. After adjustment for potential confounding factors, the differences in persistence between diuretics and ACE-inhibitors did not remain (Fig. 2).

Adjusted class persistence was lower in men than in women (*P* = 0.004), as well as in patients with young age (30–49 years) (*P* < 0.001), with lower systolic blood pressure (*P* < 0.001), and those born outside the Nordic countries (<0.001) (Table 4). We observed that the crude data for persistence in patients with diabetes were nonsignificant, and became significant after adjustment. This result is explained by a lower systolic blood pressure in patients initiated on treatment compared to patients without diabetes (data not shown).

**Table 4:**
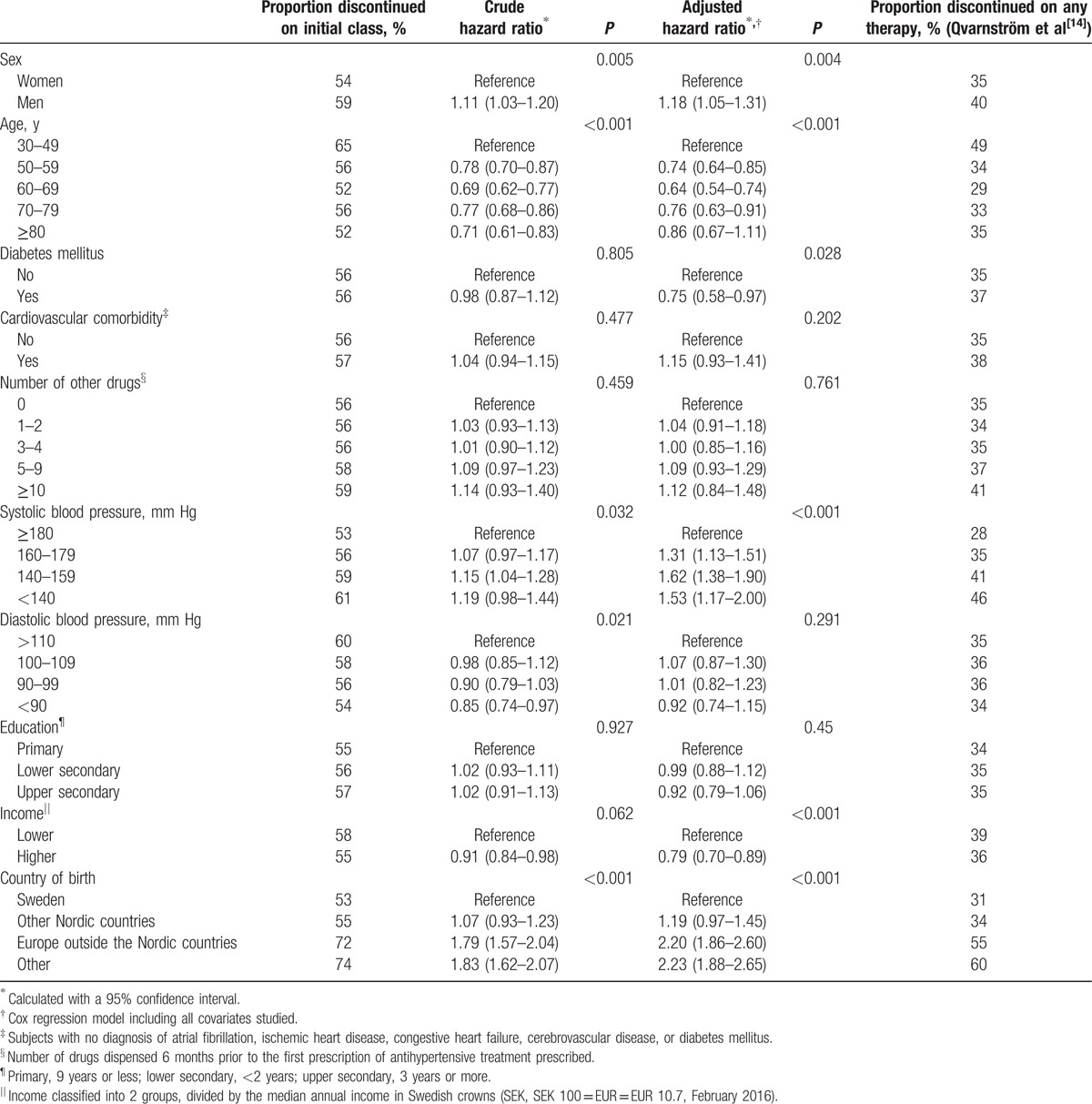
Factors associated with discontinuation of antihypertensive drug therapy 2 years.

### 3.3 Switching between drug classes

**Figure 3:**
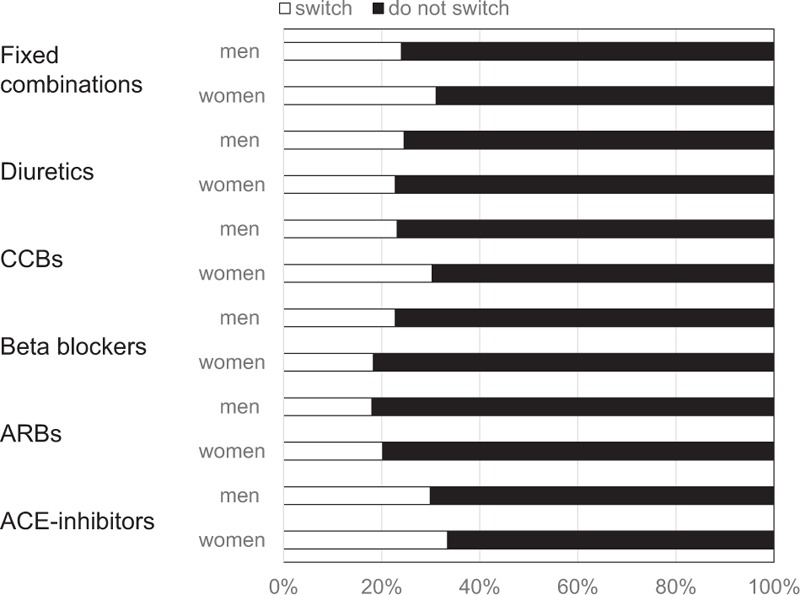
Percentage of patients who switch to another antihypertensive drug class according to treatment in 4997 patients with hypertension in primary healthcare initiated on therapy 2006 to 2007. ACE = angiotensin converting enzyme, ARB = angiotensin receptor blocker, CCB = calcium channel blocker.

Switching occurred in 1295 patients (14% women and 12% men, *P* < 0.01). The proportion of switchers was larger among patients on ACE-inhibitors and smaller for patients initiated on ARBs. The differences in the proportions of men and women who switched the new initiated drug class are illustrated in Fig. 3. Only 1 in 17 patients had a blood pressure recorded within 26 weeks before the switch occurred, and 53 (69%) of these blood pressure values were 140/90 mm Hg or higher.

## 4 Discussion

This study of 4997 patients newly initiated on antihypertensive treatment in Swedish primary healthcare have several important findings. First, more than half of the study population discontinued their initial treatment within 2 years, while 1 in 4 patients switched to another drug class during the same period. Second, class persistence was similar between diuretics and any of the other antihypertensive treatments after adjustment for potential confounders. Third, major determinants behind discontinuation of the initial drug class were male sex, lower age, a lower systolic blood pressure before initiation of treatment, and country of birth outside Sweden. Fourth, ACE-inhibitors were the most commonly filled prescription of an antihypertensive drug class, followed by diuretics and beta blockers. Finally, there were some important differences in background characteristics for patients initiated on different drug classes, such as educational level and income.

### 4.1 Drug discontinuation

One-fourth of all patients filled 1 prescription only, and approximately 40% of all patients discontinued their initial drug class during the first year. This high proportion of patients discontinuing treatment early after initiation confirms findings from other studies.^[7,9,19]^ In studies where antihypertensive drug classes were compared with 1 another, diuretics and beta blockers most often have been reported with the lowest class persistence,^[9,10,12]^ whereas ARBs have the highest class persistence.^[20–22]^ Accordingly, our crude results found a lower persistence for ACE-inhibitors than for diuretics. More important, however, this difference did not remain after adjustment for confounding factors shown to be important for drug class persistence. Thus, we found no differences in persistence between diuretics and the other drug classes. Our observations thus support recent findings on persistence from Germany also using primary healthcare population data, adjusting for many possible confounders.^[19]^ These results suggest that there are no important differences in persistence between the most common antihypertensive drug classes. Prior studies appear to have been biased by not adjusting for important variation between patients initiated on different antihypertensive treatments.

We observed lower class persistence in patients initiated on fixed drug combinations. This may be due to residual confounding, if patients who were offered a fixed combination initially had a more unfavorable cardiovascular risk profile or other comorbidities. It is also important to acknowledge the low number of patients who were offered fixed combinations, which may have caused unreliable results. However, fixed combinations might not offer the substantial benefits previously suggested.^[23]^ The present results do not offer further conclusions on this, since we did not assess combinations of antihypertensive drugs when given separately at the same time. Properly designed randomized controlled studies are needed to verify whether fixed dose combinations can offer an advantage for antihypertensive treatment in the context of class persistence.

### 4.2 Factors affecting drug class persistence

Overall, our observations on patient characteristics influencing drug class persistence are in line with most studies in the field. Thus, higher discontinuation rates in men than in women are in concordance with other studies.^[9,24,25]^ This finding supports the idea of men being less involved in their preventive care.^[26]^ We found that younger patients had lower class persistence, confirming other studies on drug class and therapy persistence.^[24]^ This may be explained if age might influence how therapeutic benefit is regarded and valued. The potential associations between socioeconomic factors and drug class persistence in hypertension treatment have been less studied. We found that patients with high income and born in Sweden had lower discontinuation rates, while no association was found between educational level and persistence. These results confirm findings from an Italian study.^[27]^ The finding of poor persistence in patients born abroad may be related to deficiencies in verbal communication between the caregiver and patient. The implications of these results are that patients with male sex, young age, mild systolic blood pressure elevation, and birth outside of Sweden should be offered extra attention from primary healthcare in order to improve their medication persistence.

### 4.3 Switching of drug classes

Approximately 1 in 4 patients switched their first antihypertensive treatment to another drug class. This may in part be due to an insufficient blood pressure reduction. Indeed, two-thirds of all patients who had a blood pressure recorded before the switch still had an uncontrolled blood pressure. However, it appears more likely that lack of blood pressure control despite treatment would lead to the addition of another antihypertensive drug class in order to improve blood pressure reduction. It is also important to acknowledge that only 1 out of 5 patients had a blood pressure recorded before the switch, suggesting that other factors were considered important for the switch. A possible explanation may be adverse drug reactions.

We found that women were more class persistent than men. It has been suggested that women more frequently experience side effects from drug treatment and more frequently consult primary care, and thus would be expected to be more inclined to switch between antihypertensive drug classes.^[26,28,29]^ However, we were not able to confirm this in a recent study.^[30]^ Thus, men appear to be at higher risk for poor persistence both to the initial drug class and to any antihypertensive therapy than women. This may imply more careful follow-up after initiation of antihypertensive drug treatment in men.

### 4.4 Initial drug class

In this study, ACE-inhibitors were the most common initial drug therapy. At the time of the study, ARBs (generically available first in 2010) were more expensive in Sweden than the generically available ACE-inhibitors, leading to several activities promoting the use of ACE-inhibitors in favor of ARB.^[31]^ Our study on newly initiated therapy extends previous findings, showing that concomitant disease conditions such as diabetes, when drugs blocking the renin–angiotensin–aldosterone system are indicated, are important when choosing antihypertensive drug class.^[32]^ We also found that beta blockers, although no more favored as first choice treatment, were initiated to one-fifth of the patients. These findings, confirming previous studies in Sweden,^[17,32]^ might at first be surprising. However, beta blockers were long common first line antihypertensive therapy in Sweden, and changes in prescribing traditions are slow. Furthermore, it is important to acknowledge the differences in background characteristics for the patients filling their first prescription of the different drugs. Thus, there was an association between educational level and income, and use of ARBs, in confirmation of previous studies.^[33]^

### 4.5 Strengths and limitations

Compared to previous investigations on medication persistence, this study has several strengths. First, we studied a large unselected primary care population of patients with hypertension from 2 Swedish regions. Second, data extraction was made independent of the caregiver or patient, and was thus not biased by any selection of patients or physicians. Third, we assessed factors seldom studied in association with persistence, for example, socioeconomic status and level of blood pressure before initiation of treatment. Fourth, all patients had a diagnosis of hypertension prior to the initiation of antihypertensive drug treatment. Antihypertensive drugs are used for other indications than hypertension, such as migraine, coronary heart disease, and heart failure. Thus, studies including patients without a conformed diagnosis of hypertension might overestimate or underestimate persistence. Fifth, we used the dosage instructions on each prescription to determine the end of the filled prescription's supply, since it is likely to provide more accurate information than aggregated data on defined daily doses, which has been used as exposure measurement in some studies.^[8,13,19]^ Thus, we believe that our results are well representative for a general population of patients with hypertension in Sweden.

There are also potential study limitations to be considered. This includes possible bias in the recordings of diagnoses and blood pressures. However, we have previously found a high validity in blood pressure measurements and in reporting a diagnosis of hypertension in this setting.^[15,34]^ Second, due to the fact that drugs to a large extent are reimbursed by the government in Sweden and several other European countries, there is a possibility that patients fill their prescription without using them. Therefore, assessing medication persistence through patients filling their prescriptions might lead to overestimation of persistence in our study.^[35]^ Third, there may also be methodological limitations in the definition of switching, with potential underestimation of switchers in our study. Fourth, we did not analyze to what extent the lack of difference in class persistence could have reflected differences in blood pressure lowering effect between the drug classes. However, only 28% of all patients had a recorded blood pressure after drug initiation, indicating that poor blood pressure control was not the key driver behind poor persistence. Fifth, reimbursement restrictions were introduced for antihypertensive drugs in Sweden in September 2008, affecting availability and prices of antihypertensives.^[17]^ Since we restricted our study to patients initiated on treatment before the restrictions were introduced we believe this has not influenced our findings. Still, it is important to acknowledge that patient expiries of ARBs and other changes on the drug market after the study was conducted may have implications on the generalizability of our findings.^[36]^ Finally, we acknowledge that our study lacked information on adverse events, quality of life, or other patient-related outcomes measure that may explain the differences in discontinuation rates.^[37]^ Such studies are urgently needed to further assess to what extent the observed differences in persistence and switching between women and men may be explained by adverse events or if differences between countries concerning the tolerability of the different drugs may have implications on generalizing our findings to countries outside Northern Europe.^[38,39]^

## 5 Conclusions

This study among patients with hypertension in primary healthcare newly initiated on drug therapy suggests that persistence is similar for any antihypertensive treatment when compared with diuretics, provided that confounding factors are properly adjusted for. Such confounding factors have often been overlooked in previous studies, where different results have been reported. Similar persistence between diuretics and other antihypertensive drug treatments inclines that the patients ought to be prescribed the most appropriate drug class according to their individual risk profile and comorbidity. Furthermore, the large number of patients discontinuing the therapy calls for improvement in how patients are monitored and motivated. Patients with male sex, young age, mild systolic blood pressure elevation, or born abroad may need extra attention to improve class persistence.
